# Intravenous Thrombolysis with Urokinase for Acute Ischemic Stroke

**DOI:** 10.3390/brainsci14100989

**Published:** 2024-09-28

**Authors:** Yue Qiao, Jing Wang, Thanh Nguyen, Lan Liu, Xunming Ji, Wenbo Zhao

**Affiliations:** 1 Department of Neurology, Xuanwu Hospital, Capital Medical University, Beijing 100053, China; 2 Department of Neurology and Radiology, Boston Medical Center, Boston, MA 02118, USA; 3 Beijing Institute for Brain Disorders, Capital Medical University, Beijing 100053, China; 4 Department of Neurosurgery, Xuanwu Hospital, Capital Medical University, Beijing 100053, China

**Keywords:** intravenous thrombolysis, urokinase, acute ischemic stroke, cerebrovascular disease

## Abstract

Background: Intravenous thrombolysis is one of the most effective therapies for the treatment of acute ischemic stroke (AIS), with urokinase offering a cost-effective alternative to newer agents like alteplase and tenecteplase, especially in resource-limited settings. Methods: This review provides a comprehensive overview of the application of intravenous thrombolysis with urokinase for AIS in the clinical practice of stroke management, including the efficacy, safety, and cost-effectiveness of urokinase compared to other thrombolytic agents. Results: Urokinase, a first-generation thrombolytic drug, is a non-specific plasminogen activator that offers a cost-effective alternative. It has been used in clinical practice for over two decades to improve neurological outcomes in patients with AIS if administered within 6 h of ictus. Numerous studies have indicated that urokinase remains a viable option for patients who cannot access alteplase or tenecteplase because of economic constraints, time window limitations, availability, or other reasons. Conclusions: In low- and middle-income countries, urokinase is a cost-effective alternative thrombolytic drug. High-level evidence-based medical research is therefore urgently needed to confirm that urokinase is not inferior to new-generation thrombolytic drugs, and to assess whether it may even be superior in some patient populations.

## 1. Introduction

Stroke is ranked as the second-leading cause of death worldwide, and the leading cause of death in China, with data in 2019 showing that ischemic strokes account for over 75% of all stroke incidents reported in China [1]. Acute ischemic stroke (AIS) can result from an arterial occlusion, and intravenous thrombolysis (IVT) is crucial to mitigate its devastating effects by recanalizing the occluded artery [2,3]. Several thrombolytic drugs, including streptokinase, staphylokinase, urokinase, recombinant tissue plasminogen activator (rt-PA), tenecteplase (TNK-tPA), and reteplase, are commonly used in clinical practice, each with unique characteristics [4,5]. Alteplase, a type of rt-PA, was approved by the U.S. Food and Drug Administration (FDA) in 1996 for the treatment of AIS [2,4]. With significant evidence indicating its efficacy when administered within the first 4.5 h of symptom onset, alteplase is widely considered the gold standard in thrombolytic treatment. However, this drug has several limitations, including a short half-life, the need to be continuously administered over 1 h, a relatively low recanalization rate, a narrow therapeutic window, the risk of hemorrhagic complications, and high cost [6]. Although alteplase remains the most widely applied agent for acute ischemic stroke, newer agents, such as tenecteplase, offer promising alternatives that may simplify administration, requiring only a single bolus rather than an infusion over an hour, and reduce complications. Further, an increasing number of new thrombolytic drugs are being developed, while their use is becoming more convenient and their safety is improving. As such, the question is whether we should use or eliminate first-generation thrombolytic drugs, which have also been proven to be an effective therapy for AIS, as recommended by the guidelines of several countries [7], and have the advantage of being cost-effective [8].

Urokinase was approved by the China Food and Drug Administration two decades ago, while IVT was recommended in Chinese guidelines within 6 h of AIS onset (Class II, Level B). It is an effective alternative to alteplase, offering a shorter treatment window of 4.5 h (Class I, Level B) [7,9]. Patients with AIS can receive intravenous urokinase thrombolysis at a dosage of 1,000,000–1,500,000 IU, supported by evidence from two trials published in China. One study included 409 patients, while the other involved 465 patients, although both had relatively small sample sizes [10,11]. Its relatively lower cost compared with alteplase makes it an attractive option in economically constrained settings [12]. Several clinical trials and meta-analyses have previously evaluated the efficacy and safety of urokinase in treating acute ischemic stroke [13,14]. Over the past several decades, urokinase has been widely investigated, highlighting its potential in improving functional outcomes and reducing mortality, albeit with only varying degrees of success. Additionally, the risk of hemorrhagic complications, a major concern in thrombolytic therapy, has been extensively investigated [15]. Understanding the intricacies of urokinase therapy has the potential to enhance clinical decision-making and refine treatment strategies for ischemic stroke, particularly in cases where cost-effectiveness is crucial.

This review aimed to provide a comprehensive overview of the current evidence on urokinase thrombolysis in ischemic stroke, examining its pharmacodynamics, clinical efficacy, safety profile, and cost-effectiveness. By synthesizing the findings from pivotal studies, including the results of early randomized trials published in Chinese, we sought to render these results accessible to English readers. We further aimed to understand the role of urokinases in contemporary stroke management and to identify areas for future research.

## 2. Pharmacological Mechanism of Urokinase

**Figure 1:**
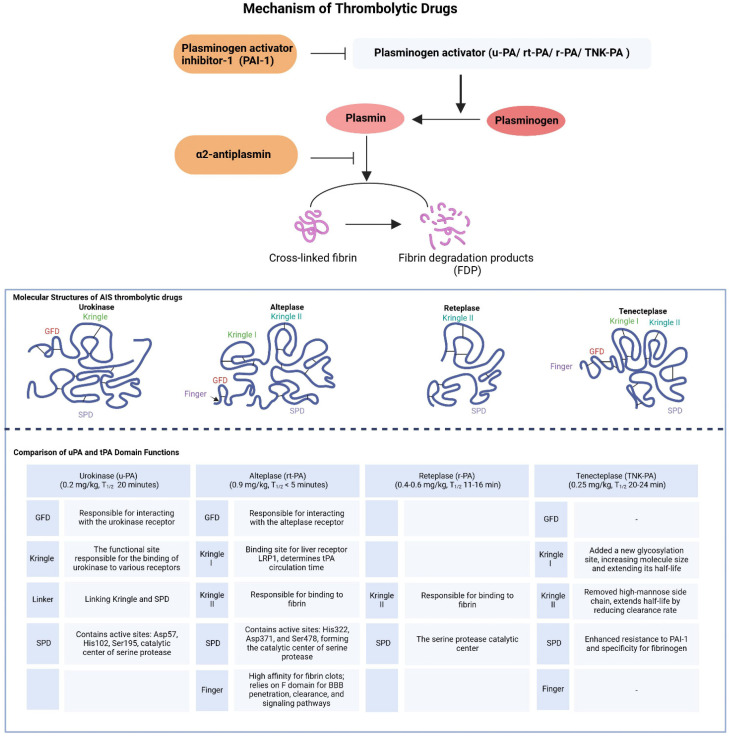
Pharmacodynamics and pharmacokinetics of thrombolytic drugs.

There are two types of plasminogen activators (PAs) in the blood: tissue plasminogen activator (t-PA), secreted by vascular endothelial cells, and urokinase plasminogen activator (u-PA). These differ significantly in their pharmacological mechanisms and pharmacokinetics (Figure 1) [16,17]. Urokinase binds to receptors on cell surfaces, concentrating its activity at thrombi, thus minimizing systemic fibrinolysis. Owing to its relatively short half-life, continuous infusion is required to maintain therapeutic levels [18].

In contrast, alteplase contains a fibronectin finger domain, an epidermal growth factor domain, two Kringle domains, and a serine protease domain. These domains enable a high specificity of fibrin, localizing its activity to thrombi [19,20]. However, alteplase is rapidly inhibited by plasminogen activator inhibitor-1 (PAI-1), forming a complex quickly cleared by the liver, resulting in a short half-life of approximately 5 min [21]. Reteplase and tenecteplase were developed from alteplase to address these limitations. Reteplase, a modified form of alteplase, has a longer half-life and increased resistance to PAI-1 inhibition, owing to deletions in its domain structure which simplify its protein configuration and enhance its pharmacokinetic profile [22]. Tenecteplase was engineered through the introduction of specific point mutations that improved its resistance to PAI-1 and extended its half-life while maintaining high fibrin specificity. These modifications allow for the administration of tenecteplase as a single bolus injection, which is more convenient than the continuous infusion required for alteplase [23,24,25]. Considering these pharmacokinetic and pharmacodynamic characteristics, urokinases and alteplases have distinct advantages and challenges, with ongoing optimization aimed at enhancing their therapeutic efficacy by learning from the structural and functional properties of different drugs.

## 3. Clinical Efficacy and Safety of Urokinase in Ischemic Stroke

Clinical trials in the subsequent decades rigorously evaluated the efficacy and safety of urokinase, and representative trials are summarized in Table S1.

### 3.1. Intravenous Thrombolytic within 6 h with Urokinase

#### 3.1.1. Early Clinical Trials and Initial Findings

The high cost of alteplase and its strict administration time window limit its widespread use, particularly in patients with limited economic resources. To find a more cost-effective alternative, the Chinese government supported research on urokinase during the “Ninth Five-Year Plan” for treating acute cerebral infarction within 6 h of symptom onset. In the first phase of the study, an open-label, single-arm study was utilized: a total of 409 patients were included, with 43 patients receiving urokinase thrombolysis within 3 h of onset, 216 patients between 3 and 6 h, and 150 patients between 6 and 12 h. The results revealed that the European Stroke Scale (ESS) scores significantly increased 2 h after thrombolysis, with some patients recovering muscle strength from paralysis grade 0 to above grade 3, and 87.5% of the patients showing an increase in the score of more than 10 points by 24 h. The symptomatic intracranial hemorrhage (sICH) rate was 3.91%, the non-symptomatic intracranial hemorrhage (nsICH) rate was 4.64%, and the 90-day mortality rate was 12.22%. These values were lower than those reported for rt-PA thrombolysis. Overall, these results suggest that urokinase was effective at treating AIS, and with strict control of the time window and indications, it was relatively safe.

In 2002, the second phase of the study was initiated using a multicenter randomized trial design. A total of 465 patients with AIS were randomly allocated: Group A received 1,500,000 U of urokinase intravenously over 30 min, Group B received 1,000,000 U of urokinase, and Group C received a placebo of normal saline [10]. The 90-day outcome showed significantly better neurological recovery in the urokinase groups compared to the placebo group, with the ESS scores at 90 days being 86.36 ± 18.98 (Group A), 84.44 ± 17.67 (Group B), and 75.43 ± 22.52 (Group C). A 90-day mRS score of 0–1 was achieved by 44.90% of the patients in Group A, 45.51% in Group B, and 31.88% in Group C, with a statistically significant difference between groups B and C (*p* = 0.028). sICH rates were 4.52% (Group A), 3.09% (Group B), and 2.03% (Group C). Notably, these findings provided early results that laid the foundation for subsequent research, and have been incorporated into the Chinese clinical guidelines for the treatment of AIS [5].

Subsequently, several small-sample studies [26,27,28,29] showed that patients with AIS who received urokinase treatment within 6 h exhibited significant improvements in NIHSS scores and clinical symptoms compared with the control group. In addition, compared to batroxobin [30], the urokinase group showed a rapid onset of efficacy within the first 6 h, as measured by improvements in the NIHSS score, whereas the batroxobin group showed more stable improvements over time. When comparing the Barthel Index (BI) and mRS 0–2 scores at three months, there were no significant differences between the two groups. However, further research is required to determine long-term outcomes beyond 90 days to better understand the efficacy and safety of urokinase in the treatment of acute cerebral infarction.

#### 3.1.2. Urokinase Dosage Comparisons and Efficacy

Given the lack of specificity of urokinase for fibrin, clinicians often use larger doses to enhance thrombolytic efficacy; however, these higher doses may elevate the risk of hemorrhagic complications. One clinical study conducted in 2016 compared the efficacy and safety of intravenous (IV) administration of 1,000,000 versus 1,500,000 units of urokinase [31]. The results indicated no significant difference in overall therapeutic efficacy between the two dosage groups; however, the incidence of adverse reactions was significantly lower in the 1,000,000-unit IV group. A subsequent study in 2020 supported these results, showing that patients receiving the lower dose had significantly better hemorheological parameters, including reduced high and low shear whole blood viscosity, plasma viscosity, and fibrinogen levels [32]. Another study indicated that medium-dose urokinase (1,000,000 IU) provided therapeutic benefits similar to those of high-dose urokinase (1,500,000 IU) for cerebral infarction treatment, as measured by NIHSS, BI, and mRS scores. However, the medium dose significantly reduced adverse reactions, suggesting that it should be preferred in clinical practice [31].

Recent studies have extensively compared early thrombolysis with urokinase treatment with conventional treatments. For example, Shao et al. revealed that the thrombolysis group had a significantly higher recovery rate of 70.5% compared to 45.2% in the control group, and the 90-day post-treatment Fugl–Meyer Assessment scores and BI were significantly improved in the thrombolysis group, with a lower incidence of adverse reactions [33]. Furthermore, other studies have demonstrated that urokinase can mitigate neural tissue damage and promote neurological function recovery by inhibiting inflammatory responses and oxidative stress [34,35]. These studies suggested that intravenous thrombolysis with urokinase is an effective and safe treatment strategy for acute cerebral infarction, significantly improving neurological outcomes and exerting neuroprotective effects. However, further large-scale clinical trials are required to confirm and determine the optimal dosage and therapeutic time window.

#### 3.1.3. Influencing Factors and Patient Characteristics

Aside from dosage, several clinical studies have highlighted other key factors influencing the efficacy of urokinase thrombolysis in AIS, including atrial fibrillation, elevated pre-thrombolysis blood glucose levels, high pre-thrombolysis ESS or NIHSS scores, and prolonged onset-to-needle time (ONT) [36,37,38,39]. One study on elderly patients with hyperacute ischemic stroke found that patients with atrial fibrillation or high blood glucose levels before thrombolysis had poorer outcomes with urokinase treatment [37]. While comparisons of the efficacy of urokinase across different age groups revealed that younger patients (<60 years) benefit more from urokinase treatment, with better neurological recovery and fewer complications compared to older patients (≥60 years), younger patients exhibited significantly lower NIHSS scores and improved mRS and Stroke Impact Scale scores at 28 and 90 days post-treatment, indicating faster and more complete patient recovery. Moreover, the incidence of complications within 72 h post-thrombolysis was significantly lower in younger patients than in older ones [40]. The location of cerebral infarcts affects prognosis, with those in the posterior cerebral artery territory generally having a better outcome than those in the middle cerebral artery territory [41].

These findings underscore the importance of patient age and other individual characteristics when determining the optimal urokinase dose and treatment plans for different patient groups. Exploring the efficacy of urokinase intravenous thrombolysis in elderly patients (aged 85 and older) is a crucial and valuable future research direction [42]. Regarding urokinase dosage, the use of medium-to-high doses (1,000,000–1,500,000 U), early administration of the drug within 3–4.5 h of onset, and treatment of younger patients with fewer underlying conditions result in better outcomes. Future research should therefore focus on refining the optimal dosage and timing for different patient subgroups and understanding the long-term outcomes of urokinase treatment compared to standard thrombolytic drugs (e.g., alteplase or tenecteplase).

### 3.2. Comparison of Urokinase and Alteplase

#### 3.2.1. Administration of Alteplase and Urokinase

Alteplase received approval and recognition as the standard thrombolytic treatment for AIS worldwide following the publication of the National Institutes of Stroke and Neurological Diseases trial in 1995 [43]. Alteplase has a high thrombolytic specificity, thus minimizing systemic effects on the coagulation system and other organs, and theoretically reducing bleeding risk. However, in clinical practice, alteplase is associated with the risk of vascular re-occlusion, and intracranial and systemic hemorrhagic complications. Furthermore, its high cost and short therapeutic window (<4.5 h) contribute to its relatively low clinical utilization rate. In later time windows (beyond 4.5 h), its use requires advanced imaging, which is limited to low- or middle-income countries [44,45].

#### 3.2.2. Comparative Clinical Outcomes of Alteplase and Urokinase

The first nationwide prospective registry study comparing urokinase and alteplase in the treatment of AIS was termed the INTRECIS study [9]. This study enrolled 3810 patients treated within 4.5 h of symptom onset, with results indicating that both variable-dose alteplase and high-dose urokinase (1.0–1.5 × 1,000,000 U/kg) achieved similar rates of excellent recovery (mRS 0–1) at 90 days (95% CI 0.98 to 1.35, *p* = 0.078) without increasing the incidence of sICH (95% CI 0.35 to 1.35, *p* = 0.281). However, in one retrospective study analyzing 111 AIS patients treated with either alteplase or urokinase, Su et al. found that the alteplase group had a complication rate of 5.97% compared to 20.45% in the urokinase group, with a lower incidence of intracranial hemorrhage (2.99% vs. 6.82%) [46]. This increased risk associated with urokinase is thought to be due to its ability to not only promote thrombolysis, but also degrade fibrinogen and coagulation factors in the circulatory system, leading to a higher risk of systemic bleeding.

Based on one sample of 54 patients, Lin found that alteplase was more effective than urokinase in treating AIS, but carries a higher risk of sICH [47]. A cohort study by Zong et al. [48] included AIS patients treated within 6 h of onset registered in the Chinese Stroke Center Alliance (CSCA) from January 2016 to December 2022. Among these patients, 113,521 underwent alteplase intravenous thrombolysis and 30,950 underwent urokinase intravenous thrombolysis. The results showed that patients receiving urokinase intravenous thrombolysis had a lower risk of hemorrhagic transformation following cerebral infarction and in-hospital death than those receiving alteplase; however, there was no significant difference in the rate of good functional outcomes at discharge between the two groups.

In summary, although both urokinase and alteplase represent effective treatments for AIS, urokinase may offer a lower risk of hemorrhagic transformation and in-hospital mortality, although it should be noted that these patients had higher overall complication rates. However, a key limitation in this area of research is the lack of high-quality, randomized controlled trials directly comparing urokinase to newer thrombolytic drugs.

#### 3.2.3. Long-Term Efficacy and Safety

According to one in vitro study, alteplase has a greater affinity for the thrombus and results in faster thrombolysis than urokinase [19]. However, this advantage diminishes, particularly in the later stages of treatment, as the initial targeted action of alteplase diminishes. One retrospective study conducted by Su et al. found that the NIHSS scores in the alteplase group were significantly lower than those in the urokinase group at 3, 24, 7, and 2 weeks post-treatment. However, there was no significant difference between the groups in terms of the 3-month functional outcome, as evaluated by mRS [46]. Chen et al. further conducted a secondary analysis of elderly (≥75 years) AIS patients from the INTRECIS cohort. They found no significant differences between the alteplase and UK groups in terms of 90-day functional outcomes (mRS 0–1), or changes in NIHSS scores at 1 and 14 days post-treatment. However, alteplase has been associated with lower overall mortality and a significantly reduced mortality in patients with atrial fibrillation [49].

In one 2019 study, patients with AIS were grouped based on the time from symptom onset to IVT, and then treated with either urokinase or alteplase. Both alteplase and urokinase were found to be equally effective and safe in acute ischemic stroke, with earlier treatment yielding better outcomes [50,51]. Although alteplase is often preferred because of its targeted action and established protocols, urokinase is a valuable alternative, particularly in settings where cost and availability are major considerations. Further research is required to refine the treatment strategies and improve patient outcomes in diverse clinical contexts.

### 3.3. Urokinase in Combination with Anticoagulation or Antiplatelet Therapy

Research has shown that thrombolysis may increase thrombin activity and decrease plasmin activity in patients with AIS, leading to new blood clots [52]. As such, anticoagulation or antiplatelet therapy following IVT is an important therapy to prevent thrombogenesis and vessel re-occlusion, even though they carry the potential risk of bleeding. In a 2015 retrospective study [53], 126 patients receiving arterial or intravenous urokinase thrombolysis were divided into two groups: one group received low molecular weight heparin within 6 h post-thrombolysis, and a control group received anticoagulation therapy 24 h after thrombolysis. Additionally, one recent study showed that tirofiban administered within 30 min after urokinase IVT improved neurological function and daily living activities, achieved higher overall clinical efficacy, and significantly improved platelet function indicators compared to urokinase thrombolysis alone [54,55]. It was also observed that the NIHSS score on day 7 and the mRS score at 3 months were lower in the tirofiban combined with urokinase group than in the urokinase alone group. However, there was no significant difference between the two groups in terms of the incidence of sICH within 7 days of admission, or in the mortality rate within 3 months [56]. These findings suggest that tirofiban is a potentially safe adjunctive treatment for patients with AIS undergoing urokinase thrombolysis, with the capability to improve short-term neurological outcomes.

### 3.4. Urokinase Thrombolysis Followed by Endovascular Therapy

Bridging therapy with endovascular thrombectomy following intravenous thrombolysis is commonly applied in patients with AIS. Recent studies have shown that combining urokinase thrombolysis with Solitaire AB stent thrombectomy is an effective treatment for acute anterior circulation large artery occlusion, achieving high recanalization rates and favorable outcomes with low complication rates [57,58,59]. Additionally, one study evaluating the efficacy and safety of urokinase bridging therapy in patients with AIS administered within 4.5–6 h of onset included 47 patients who were randomly divided into a urokinase bridging therapy group and a urokinase intravenous thrombolysis group revealed that the bridging therapy group had a significantly higher rate of good prognosis at 90 days (44%) than the intravenous thrombolysis group (17%) (*p* = 0.045). The incidence of sICH was also slightly higher in the bridging therapy group (9%) compared to the intravenous thrombolysis group (4%) (*p* = 0.05), although with similar mortality rates (9% vs. 8%, *p* = 0.97) [60]. Another study showed that mechanical thrombectomy and intravenous thrombolysis bridging mechanical thrombectomy were consistent for acute large vessel occlusion stroke within 6 h of onset, and the safety and prognosis of urokinase bridging therapy were comparable to alteplase bridging therapy [61]. These findings indicate that urokinase bridging therapy administered within 4.5 to 6 h of onset provides better clinical outcomes than urokinase alone, without significantly increasing the risk of hemorrhage or death.

## 4. Time and Economic Benefits

### 4.1. Therapeutic Window and Efficacy

Currently, there is no consensus regarding the optimal time window for safe thrombolytic therapy, particularly when advanced imaging is used. Chinese guidelines recommend intravenous thrombolysis with urokinase within 6 h of symptomatic ictus; however, several early studies found that patients who received delayed urokinase thrombolysis treatment (6–24 h post-onset) also showed significant benefits [62,63,64,65]. Some researchers further noted that urokinase thrombolysis was more effective in the extended window group than in the conventional treatment group, with similar efficacies to the early thrombolysis group (within 6 h) and without severe side effects [62]. This finding was confirmed in a 2014 study, which indicated that while delayed thrombolysis provides significant benefits, it is further associated with an increased risk of hemorrhage [63]. IVT with urokinase for the treatment of acute cerebral infarction is highly effective, with research showing that administration of urokinase within 3 h of the onset of acute cerebral infarction shows even better outcomes, significantly reducing the degree of neurological damage in patients [66]. Overall, evidence suggests that extending the therapeutic window for urokinase thrombolysis in AIS beyond the traditional 3 to 6 h can be effective and relatively safe. This extended window of up to 12 h provides significant benefits without markedly increasing mortality, although it is associated with a higher risk of hemorrhagic complications. The use of advanced imaging and individualized patient assessment may be key in maximizing the benefits and minimizing the risks associated with this treatment approach.

### 4.2. Cost-Effectiveness and Accessibility

The use of IVT for AIS shows wide geographic variation, ranging from 10% to 15% in high-income nations, and less than 2% in low- and middle-income countries. In particular, the high cost of alteplase may be a major barrier to IVT in low- and middle-income countries [67].

Compared to other thrombolytic agents, such as alteplase and tenecteplase, urokinase offers significant cost advantages, making it a valuable option, particularly in regions with limited economic resources. The cost of alteplase ranges from USD 2200 to 2400 per 100 mg. It is made using advanced technology in mammalian cell culture, which is a complex and costly process [68], while its production requires high biosafety levels and strict purification protocols. In addition, alteplase is sensitive to temperature and requires specific storage conditions, which increases its overall cost [69]. Tenecteplase is a genetically engineered variant of alteplase produced using recombinant DNA technology in mammalian cell cultures, involving the insertion of the gene responsible for producing tenecteplase into the DNA of host cells, typically Chinese Hamster Ovary (CHO) cells [70]. Tenecteplase is slightly less expensive than rt-PA, costing approximately USD 2000 per dose. Further, due to its ease of administration, it is increasingly used to treat stroke [68]. In contrast, urokinase costs approximately USD 140 per 250,000 U. Urokinase is extracted from human urine, or produced using recombinant technology, involving simpler and lower-cost processes, and is predominantly used in countries with lower healthcare budgets due to its improved cost-effectiveness.

Given the United States GDP per capita of approximately USD 76,330, the use of alteplase and tenecteplase is feasible and widespread despite their high costs. However, for developing countries such as India (GDP per capita in 2022: USD 2411), China (GDP per Capita in 2022: USD 12,720), and Ghana (GDP per capita in 2022: USD 2250), urokinase offers a more affordable alternative for thrombolytic therapy [71]. Given China’s economic context, where a significant portion of the population has relatively low income levels, using urokinase for thrombolytic therapy is particularly suitable. According to recent statistics, around 600 million individuals in China earn a monthly income of approximately RMB 1000 (i.e., approximately USD 137), highlighting the financial challenges that many families encounter [72]. This economic reality indicates that cost-effective medical treatments, such as urokinases, are more accessible and feasible for a large segment of the population.

## 5. Conclusions

The safety and efficacy of urokinase for IVT in patients with AIS have been widely investigated. Although alteplase remains the gold standard and the only globally licensed systemic reperfusion therapy, urokinase offers a viable, cost-effective alternative, demonstrating comparable efficacy in improving neurological outcomes within a 6-h treatment window. In the past 20 years, the successful use of urokinase thrombolysis for AIS has provided a successful example of IVT treatment of AIS in low- and middle-income countries. However, this focus may limit the applicability of the results to other populations, and the urokinase-related studies included show heterogeneity in patient selection criteria, treatment protocols, and outcome measures. For patients who cannot access alteplase treatment owing to economic constraints, time window limitations, hospital capabilities, or other reasons, urokinase is a viable alternative for thrombolytic therapy. Thus, thrombolytic therapy with urokinase is likely to be superior to no thrombolytic therapy. The optimal noninferiority margin may accept the efficacy of intravenous urokinase compared with alteplase or tenecteplase, which is not well established. However, further randomized trials are needed to confirm the noninferiority of urokinase to alteplase or tenecteplase, as well as the safety and efficacy of urokinase for patients after 6 h of ictus.

## Supplementary Materials

The following supporting information can be downloaded at: https://www.mdpi.com/article/10.3390/brainsci14100989/s1, Table S1: Representative clinical studies of IVT with Urokinase for Acute Ischemic Stroke in China. References [10,11,12,27,28,29,30,31,33,35,39,46,48,49,50,51,58,59,61,63,64,65,66] are cited in the Supplementary Materials.
