# Supplementary material for: Intravenous Thrombolysis with Urokinase for Acute Ischemic Stroke
Source: Brain Sci. 2024 Sep 28;14(10):989. doi: 10.3390/brainsci14100989 (PMC11505899; doi:10.3390/brainsci14100989)
Supplement: Supplementary file 1 [file brainsci-14-00989-s001.zip › brainsci-3206007-supplementary.pdf]

Supplementary materials

Search strategy and selection criteria

Data for this Review were identified by searches of CNKI, WanFang Data, PubMed, and Web of Science, and references to relevant articles using the search terms "urokinase", "acute ischemic stroke", "acute cerebral infarction", and "Intravenous thrombolysis". Due to the large number of urokinase-related clinical studies published in Chinese journals, articles published in both Chinese and English between 1994 and 2024 were included.

The inclusion criteria for this review were original research articles, randomized controlled trials (RCTs), observational studies, systematic reviews, and meta-analyses that reported on the efficacy, safety, and clinical outcomes of intravenous urokinase therapy in acute ischemic stroke patients. Review articles, case reports, letters, editorials, and conference abstracts were excluded. For studies that included overlapping patient cohorts, the most comprehensive or recent study was selected to avoid duplication of data.

The Table S1 included a total of 23 studies. Data extraction was independently conducted by two authors, with any discrepancies resolved through discussion or by the corresponding author. The primary outcomes of interest included clinical efficacy (as measured by scales such as NIHSS and mRS), safety (particularly the incidence of symptomatic intracranial hemorrhage).

Table S1. Representative clinical studies of IVT with Urokinase for Acute Ischemic Stroke in China

Study	Unit	Study design	Patients	Sample Size	Intervention	Study Time	Outcomes	Results
Intravenous Thrombolysis with Urokinase within 6 Hours								
Chen et al., 2001[e1]	National 'Ninth Five-Year Plan' Key Research Project Collaboration Group	An open-label dose-finding study	AIS within 6h or PIS within 12h (age 35-80 years); carotid territory ischemia; limb strength 0-3.	15 centers, 516 cases	500,000-1,500,000 Urokinase (n=409)	1996.11-1998.6	ESS (2h, 24h, 2d, 3d, 7d, e1d, 30d, 90d), sICH, nsICH, mortality rate	(1) 24 hours, 87.5% of patients had an ESS score increase of ≥10 points.<br>(2) At 90 days, 46.6% of patients achieved an ESS score of ≥95.<br>(3) The increase in ESS scores was faster for the <3h window than the 3-6h window and faster for the 3-6h window than the >6h window. By 90 days, no significant differences were observed.<br>(4) The rate of nsICH was 4.64%.<br>(5) The rate of sICH was 3.91%.<br>(6) The mortality rate was 12.22%, with 6.35% due to massive cerebral infarction and 1.90% due to intraparenchymal hemorrhage.
Chen et al., 2002[e2]	National 'Ninth Five-Year Plan' Key Research Project Collaboration Group	RCT	AIS within 6h (age 35-75 years); carotid territory ischemia; limb strength 0-3.	51 centers, 511 cases	Group A: 1,500,000U urokinase (n=155) Group B: 1,000,000U urokinase (n=162) Group C: Placebo (n=148)	1998.7-2000.12	Primary outcome: ESS (2h, 6h, 24h, 2d, 3d, 7d, 14d, 30d, 90d)<br>Secondary outcomes: mRS, hemorrhage, and mortality rate.	(1) At 90 days, the mRS 0-1 rates were 44.90% (Group A), 45.51% (Group B), and 31.88% (Group C) (p=0.002).<br>(2) ESS scores rapidly improved within 24 hours, with significant differences observed at 90 days.<br>(3) The sICH rates were 4.52% (Group A), 3.09% (Group B), and 2.03% (Group C) (p>0.05).<br>(4) The mortality rates were 9.14% (Group A), 10.73% (Group B), and 6.10% (Group C).
Liu et al., 2003[e3]	Dalian Friendship Hospital	RCT	AIS within 6h; no low-density changes corresponding to neurological deficits.	Single Center, 76 cases	Intervention: Urokinase 250,000U IV bolus followed by 1,500,000U	2002	NIHSS (24h, 7d, 14d, 28d), overall response rate	(1) In the urokinase group, there was a significant improvement in NIHSS when comparing pre-treatment to 24 hours post-treatment, 7d to 14d, and 14d to 28d (p<0.01). Comparing 24 hours post-treatment to 7d, the

					IV infusion over 60 minutes (n=38)<br>Control: Placebo (n=38)			difference was significant ($p < 0.05$).<br>(2) The overall response rates in the Urokinase group were 24.3% at 24 hours, 57.1% at 1 week, 81.3% at 2 weeks, and 90.6% at 4 weeks ($p<0.01$).
Ling et al., 2007[e4]	The National 10th Five-Year Key Research Project	RCT	AIS within 6h (age 30-80 years); carotid and vertebral artery ischemia; limb strength 0-3.	Single Center, 102 cases	Intervention: 750,000-1,500,000U urokinase (n=52)<br>Control: Placebo (n=50)	2002.8-2007.5	NIHSS (24h, 3d, 7d, 12d, 21d), overall response rate	(1) The overall response rate of the urokinase group in the 21-day clinical evaluation was 94%.<br>(2) The mortality rates were 3.85% (Urokinase group) versus 0% (Placebo group).<br>(3) No cerebral hemorrhage or other bleeding sites were observed.
Guan et al., 2007[e5]	Harbin First Hospital Neurology Department	Retrospective Study	AIS within 6h (age 43-75 years); limb strength 0-3.	Single Center 180 cases	Intervention: 500,000-1,000,000U Urokinase (n=120)<br>Control: Standard care (n=50)	2002.1-2005.4	NIHSS (3d,5d,14d), overall response rate, fibrinogen level	(1) At 14 days, the NIHSS scores were 6.02±5.63 (Urokinase group) versus 14.44±7.11 (Control group) ($p<0.001$).<br>(2) There was a significant difference in fibrinogen levels before and after the first thrombolysis ($p<0.01$).<br>(3) The overall response rates were 83.33% (Urokinase group) versus 50.00% (Control group) ($p<0.01$).
Liu et al., 2014 [e6]	Henan Shangqiu First People's Hospital Neurology	Retrospective Study	Neurological deficits persisted for 0.5 to 4.5 hours (age 18-80 years); no low-density changes corresponded to neurological deficits.	Single Center, 136 cases	Intervention: 1,000,000-1,500,000Urokinase (n=68)<br>Control: Standard care (n=68)	2010.1-2013.12	NIHSS (24h,7d,14d), overall response rate	(1) The NIHSS scores were: 24h: 7.1±3.64 vs. 10.57±3.54, 7d: 4.93±3.35 vs. 8.85±3.53, 14d: 4.18±3.39 vs. 7.91±3.48 (all $p<0.01$).<br>(2) The overall response rate was 95.6% in the urokinase group compared to 88.2% in the control group ($p<0.05$).<br>(3) Incidence of cerebral hemorrhage: 4.41% (treatment) vs. 2.94% (control).
Li 2017 [e7]	The Fourth Affiliated Hospital of Dali	Retrospective Study	AIS within 6 hours (age 18-80 years), patients had no intracranial hemorrhage symptoms; the signs of brain function impairment persist for	Single Center, 198 cases	Urokinase 1,000,000 U (for body weight ≤ 60 kg) or 1,500,000 U (for body weight > 60 kg) dissolved	2006.1-2014.4	NIHSS, overall response rate	(1) The overall response rate for each TOAST subtype was: LAA 93.65%, CE 34.21%, SAO 92.85%, SOE 76.92%, and SUE 80.00%.<br>(2) The NIHSS scores for LAA and SAO showed significant improvement before and after thrombolysis ($P < 0.05$).

	University		more than 1 hour.		in 100 mL of saline, administered via intravenous infusion over 0.5 hours.<br>Large-artery atherosclerosis (LAA): 63 cases<br>Cardioembolism (CE): 38 cases<br>Small-vessel occlusion (SAO): 56 cases<br>Stroke of other determined etiology (SOE): 26 cases<br>Stroke of undetermined etiology (SUE): 15 cases			(3) The NIHSS scores for SOE, SUE, and CE showed significant differences when compared to 3 weeks post-thrombolysis (P < 0.05).<br>(4) No significant differences in NIHSS scores were observed for different doses of urokinase across TOAST subtypes before and after thrombolysis (P > 0.05).<br>(5) Bleeding cases were as follows: LAA 1 cases, SAO 1 cases, CE 10 cases, SOE 1 cases, and SUE 1 cases
Huang et al., 2019 [e8]	Lu Feng Shi Jia Zi People's Hospital, Guangdong	RCT	AIS within 6h	Single Center 200 cases	Intervention: 1,000,000U urokinase (n=100)<br>Control: Aspirin+ Xueshuantong (n=100)	2017.1-2018.1	NIHSS, overall response rate	(1) At 10 days, the NIHSS score was 7.2±2.1 in the urokinase group compared to 14.2±2.6 in the control group (p<0.05).<br>(2) The overall response rate was 96% in the urokinase group compared to 74% in the control group (p<0.05).<br>(3) The incidence of adverse events was 6% in the urokinase group compared to 24% in the control group (p<0.05).
Zheng et al., 2022 [e9]	Ankang Third People's Hospital	RCT	AIS within 6h (age 45-75 years).	Single Center, 60 cases	Intervention: 1,000,000-1,500,000U Urokinase (n=30)<br>Control: Standard care	2020.1-2021.4	24h NIHSS, PV, Fbg, CRP, IL-8, the overall response rate	(1) At 24 hours, the NIHSS score improved from 13.10 to 6.88 in the urokinase group compared to 12.91 to 8.41 in the control group (p<0.05).<br>(2) The overall response rate was 90% in the urokinase group compared to 66.67% in the control group (p<0.05).

					(n=30)			(3) There was a significant improvement in biochemical markers in the urokinase group compared to the control group.
Wang 2022 [e10]	First People's Hospital of Jinzhou District, Dalian	Retrospective Study	AIS within 6h (age 31-85 years).; exclude patients with cerebral hemorrhage	Single center 231 cases	Intervention: 1,000,000-1,500,000U urokinase (n=120) Control: Standard care (n=30)	2018.1-2020.12	NIHSS (3d, 7d); mRS	(1) At 3d and 7d, the NIHSS scores were (8.21±0.75) and (4.69±0.65) respectively, both significantly lower than those in the standard treatment group, which were (10.28±1.56) and (7.95±0.79) (both p<0.05) (2) At 30 days, the mRS 0-1 rate was 88.33% in the urokinase group compared to 74.77% in the control group (p<0.05).
Comparative Studies on Intravenous Thrombolysis with Urokinase and rt-PA for Acute Ischemic Stroke								
Su et al., 2017 [e11]	Yunfu People's Hospital	Retrospective study	Alteplase: thrombolysis within 4.5 hours; Urokinase: within 6 hours(age 18-80 years); NIHSS≥4； CT showed no hemorrhage.	Single Center, 111 cases	Intervention: rt-PA (n=67) Control: 1,000,000-1,500,000U Urokinase (n=44)	2014.3-2017.3	Primary outcomes: NIHSS(3h,24h,3d,7d) and intracranial hemorrhage Secondary outcomes: mRS, fibrinogen level, complication rate	(1) At 90 days, the mRS 0-2 rate was 62.69% in the rt-PA group compared to 63.64% in the urokinase group (p>0.05). (2) The sICH rate was 2.99% in the rt-PA group compared to 15.91% in the urokinase group (p<0.05). (3) At 7 days, the NIHSS score was 7.85±8.17 in the rt-PA group compared to 9.02±10.19 in the urokinase group (p<0.05). (4) The mortality rate was 1.49% in the rt-PA group compared to 6.82% in the urokinase group (p>0.05). (5) In the rt-PA group, there were 2 cases (2.99%) of fibrinogen levels dropping below 0.7g/L fewer than the 7 cases (15.91%) in the urokinase group (p<0.05).
Zhou et al., 2019 [e12]	Chifeng City Hospital	RCT	All patients met the diagnostic criteria for acute cerebral infarction; AIS within 6h (age 43-75 years); CT showed no hemorrhage.	Single Center, 98 cases	Intervention: Urokinase + rt-PA (n=49) Control: rt-PA (n=49)	2016.4-2018.1	Primary outcome: NIHSS, Secondary outcomes: sICH, nsICH, TEG, DAPKl, NO, ET	(1) Post-treatment NIHSS was 4.9±1.86 in the combination group (Urokinase + rt-PA) compared to 5.58±1.98 in the rt-PA group (p<0.05). (2) The sICH rate was 2.04% in the combination group compared to 6.12% in the rt-PA group. (3) The combination group improved oxidative stress markers and vascular

								endothelial function better but a higher incidence of cerebral hemorrhage with no significant difference in recurrent cerebral infarction and mortality.
Gao et al., 2019 [e13]	Chongqing Medical University Yongchuan Hospital	Retrospective study	All patients met the diagnostic criteria for acute cerebral infarction; Symptoms or signs duration not limited or lasting over 24 hours (if no responsible lesion on imaging); exclude non-vascular causes	Single Center 143 cases	rt-PA (n=79) ; 1,000,000U Urokinase (n=64)	2018.1-2018.12	NIHSS(24h, 7±2d), complication rate;	(1) At 7±2 days, the NIHSS score was 7.85±8.17 in the rt-PA group compared to 9.02±10.19 in the urokinase group (p<0.05).<br>(2) There was no significant difference in efficacy and complications between rt-PA and urokinase (p>0.05).
Wang et al., 2021 [e14]	INTRECIS (NCT02854592)	Nationwide Prospective Registry Study	Patients (age ≥18 years) with CT or MRI confirmed AIS who were previously well (modified Rankin scale (mRS) scores 0 or 1) and were eligible for treatment with either intravenous r-tPA or UK within 4.5hours of a definite time of onset of symptoms.	Multiple Center, 3810 cases	Variable-dose rt-PA (n=2666); High-dose urokinase (1.0–1.5×1,000,000 U/kg) (n=1144)	2017.4-2019.7	Primary outcomes: mRS and sICH;<br>Secondary outcomes: NIHSS, other bleeding events, recurrent stroke at 90 days, all-cause mortality at 90 days	(1) At 90 days, the mRS 0-1 rate was 71.3% in the rt-PA group compared to 69.5% in the urokinase group (p>0.05).<br>(2) At 90 days, the mRS 0-2 rate was 81.5% in the rt-PA group compared to 79.5% in the urokinase group (p=0.036).<br>(3) The incidence of sICH was not significantly different between the rt-PA and urokinase groups.<br>(4) The ΔNIHSS score at 24 hours and 14 days had p-values greater than 0.05.<br>(5) Intracranial hemorrhage rates were 1.1% for rt-PA and 1.4% for urokinase (p>0.05).<br>(6) Other bleeding complication rates were 1.1% for rt-PA and 0.7% for urokinase (p>0.05).<br>(7) The rate of recurrent strokes at 90 days was 1.4% for rt-PA and 2.6% for urokinase (p=0.028).<br>(8) All-cause mortality at 90 days was 4.6% for rt-PA and 5.1% for urokinase (p>0.05).

Zong et al., 2023 [e15]	Chinese Stroke Center Alliance	Cohort	AIS within 6h (age ≥18 years).	31 centers 555,991 cases	Intervention: rt-PA (n=113,521)<br>Control: Urokinase (n=30,950)	2016.1-2022.12	Primary outcome: mRS<br>Secondary outcomes: hemorrhagic transformation and mortality	(1) The discharge mRS 0-1 rate was 60.66% in the rt-PA group compared to 62.43% in the urokinase group.<br>(2) The sICH rate was 2.45% in the rt-PA group compared to 1.41% in the urokinase group.<br>(3) The mortality rate was 6.10% in the rt-PA group compared to 4.22% in the urokinase group.<br>(4) Urokinase was associated with a lower risk of hemorrhagic transformation and in-hospital death than rt-PA, but there was no significant difference in good functional outcomes (P=0.57).
Ning et al., 2023 [e16]	The National Key Research and Development Program	Retrospective study	AIS within 4.5h (age ≥78 years); mRS≤1; All patients met the diagnostic criteria for acute cerebral infarction;	60 centers, 692 cases	Intervention: rt-PA (n=509)<br>Control: Urokinase (n=183)	2017.4-2019.7	Primary outcome:90d mRS 0-1; Secondary outcome: 90d mRS 0-2; NIHSS(1d,14d); sICH, mortality.	(1) At 90 days, the mRS 0-1 rate was 71.4% in the rt-PA group compared to 64.6% in the urokinase group (p>0.05).<br>(2) At 90 days, the mRS 0-2 rate was 81% in the rt-PA group compared to 72.1% in the urokinase group (p>0.05).<br>(3) At 14 days, the NIHSS score was 4±5.4 in the rt-PA group compared to 2.5±7.8 (p<0.05).<br>(4) The mortality rate was 2.0% in the rt-PA group compared to 9.5% in the urokinase group.<br>(5) In elderly patients (≥ 75 years), rt-PA and urokinase showed similar efficacy and safety.
Urokinase Thrombolysis Beyond Time Window								

Gao et al., 2014 [e17]	Suixi People's Hospital	Open Clinical Study	AIS within 6h or 12h; all patients underwent CT or MRI to exclude hemorrhage or other diseases.	Single Center 358 cases	Intervention: 50,000-1,000,000U urokinase (n=178)<br>Control: Standard care (n=180)	2010.1-2013.12	NIHSS (6h,24h,72h,7d,14d), hemorrhage, overall response rate	(1) At 14 days, the NIHSS score was 11.72±12.15 in the urokinase group compared to 15.65±14.31 in the control group (p<0.05).<br>(2) The mortality rate was 5.7% in the urokinase group compared to 0.9% in the control group.<br>(3) The overall response rate in the urokinase group was 89.89%, but the cerebral hemorrhage rate was higher at 6.74% (p=0.032).<br>(4) Urokinase within 12 hours of acute cerebral infarction is effective but increases hemorrhagic complications; mortality remains low.
Huang 2021 [e18]	Guiping People's Hospital	RCT	All patients met the diagnostic criteria for acute cerebral infarction; AIS within 6h-24h.	Single Center 96 cases	Intervention: 30-50 U urokinase IV over 30 min + 50 U urokinase IV for 1 day (n=48)<br>Control: Standard care (n=48)	2016.12-2020.11	NIHSS(1h,24h,7d,14d), ADL, overall response rate	(1) At 14 days, the NIHSS score was 6.41±0.62 in the urokinase group compared to 7.98±0.96 in the control group (p<0.05).<br>(2) The mortality rate was 6.74% in the urokinase group compared to 0.9% in the control group.<br>(3) The ADL score at 14 days was 67.87±6.53 in the urokinase group compared to 61.16±6.02 in the control group (p<0.001).<br>(4) The overall response rate was 93.75% in the urokinase group compared to 79.17% in the control group (p<0.05).
Comparison of Urokinase Thrombolysis with Anticoagulant or Antiplatelet Therapy								
Huang 2006 [e19]	The National 10th Five-Year Key Research Project	RCT	AIS within 6h	12 centers, 74 cases	Group A: Urokinase (n=26) Group B: regular dose batroxobin (n=25) Group C: high dose and extended course batroxobin (n=23)	2002-2003	Primary outcome: NIHSS; Secondary outcome: mRS and mortality	(1) At 14 days, the NIHSS scores were 6.05±5.83 in the urokinase group, 6±5.64 in the regular dose batroxobin group, and 6.7±4.78 in the high dose batroxobin group.<br>(2) At 90 days, the mRS 0-2 rates were 42.3% in the urokinase group, 14.7% in the regular dose batroxobin group, and 42.9% in the high dose batroxobin group (p>0.05).<br>(3) The mortality rates were 15.38% in the urokinase group, 12% in the regular-dose batroxobin group, and 0% in the high-dose batroxobin group.<br>(4) Urokinase improved NIHSS faster within 2 hours but fluctuated, while

								batroxobin showed gradual and stable improvement.
Wang et al., 2024 [e20]	Wuhan Red Cross Hospital	RCT	AIS within 4.5h (age 60-80 years)	Single Center,96 cases	Intervention: Tirofiban combined with urokinase intravenous thrombolysis (n=54)<br>Control: Single urokinase intravenous thrombolysis (n=42)	2019.6-2022.9	Overall response rate, Platelet-related parameters (PDW, PCT, PAdT, PAgT), NIHSS score, Barthel index and complications	(1) The total clinical effective rate in the observation group was 96.30%, higher than 76.19% in the control group (P<0.05).<br>(2) After treatment, the levels of PDW, PAdT, and PAgT were decreased and the level of PCT was increased in both groups compared with those before treatment, with greater changes in the observation group (P<0.05).<br>(3) The NIHSS scores were decreased and the Barthel index was increased in both groups compared with those before treatment, with the NIHSS score lower and the Barthel index higher in the observation group than in the control group (P<0.05).<br>(4) There was no significant difference in the total incidence of adverse reactions between the two groups after treatment (P>0.05).
Urokinase Thrombolysis Followed by Endovascular Therapy								
Wu et al., 2017 [e21]	Ordos City Center Hospital	Case Series	AIS is due to acute anterior circulation large artery occlusion within 6 hours (age 18-80 years); it is eligible for urokinase thrombolysis and Solitaire AB stent thrombectomy.	Single Center, 8 cases	Urokinase 500,000U IV bolus followed by 1,000,000U IV infusion over 60 minutes combined with Solitaire AB stent thrombectomy (n=8).	2015.10-2016.4	Recanalization rate (mTICI), NIHSS, mRS, complications	(1) At 3 months, 6 patients had a good prognosis (mRS 0-2), 1 patient had a disability due to post-infarction hemorrhage (mRS 3-4), and 1 patient died from a large area cerebral infarction.<br>(2) The NIHSS score median at discharge was 6 (range 0-12), significantly lower than the preoperative median of 16.5 (range 12-24) (p<0.05).<br>(3) All 8 patients achieved successful recanalization with mTICI grades of 2b or 3.<br>(4) No permanent complications related to the procedure occurred.<br>(5) Urokinase combined with Solitaire AB stent thrombectomy provides a high recanalization rate and good clinical outcomes. Still, the effect on terminal ICA occlusion is less favorable, with a higher mortality rate.
Wang et al., 2022	The First District,	Retrospective Study	Acute large artery occlusion stroke patients with ineffective intravenous	Single Center,	Intravenous thrombolysis with rt-PA or urokinase	2017-5-2022.5	Vascular recanalization rate, distal blood flow recovery,	(1) The vascular recanalization rate was 81.48%.<br>(2) The distal blood flow recovery rate was 74.07%.

e22	Central Hospital of Benxi		thrombolysis using alteplase or urokinase, symptoms persisting for 3 to 4.5 hours (age≥18 years), no intracranial hemorrhage as confirmed by MRI or CT imaging.	60 cases	followed by conventional treatment for those with ineffective thrombolysis (n=27).		NIHSS scores at 2 and 4 weeks, Barthel scores	(3) At 2 and 4 weeks post-treatment, NIHSS scores were: 2 weeks: 15.98±2.57, 4 weeks:11.13±2.19.<br>(4) Barthel scores were: 2 weeks: 21.44±3.57, 4 weeks: 24.25±3.38.
Nong et al., 2022 e23	People's Hospital of Guigang	Retrospective study	Acute large vessel occlusion stroke within 6 hours of onset; eligible for mechanical thrombectomy per Chinese guidelines (2019 revision); confirmed large vessel occlusion (internal carotid artery, middle cerebral artery, vertebral artery, or basilar artery) by CT angiography or cerebral angiography.	Single Center, 105 cases (64 direct mechanical thrombectomy; 41 bridging therapy)	Group A: alteplase bridging therapy group (n=26),<br>Group B: urokinase bridging therapy group (n=15).	2019.11-2020.12	Primary outcomes: intracranial hemorrhage, mortality and mRS<br>Secondary outcomes: mTICI, NIHSS scores at 24h, nsICH	(1) At 90 days, the mRS 0-2 rate was 65.38% in the rt-PA group compared to 60% in the urokinase group.<br>(2) The rate of sICH was 15.38% in the rt-PA group compared to 20% in the urokinase group ($p > 0.05$).<br>(3) The mortality rate was 15.38% in the rt-PA group compared to 13.33% in the urokinase group ($p > 0.05$).<br>(4) The successful reperfusion rate was 92.31% in the rt-PA group compared to 93.33% in the urokinase group ($p > 0.05$).<br>(5) The 24-hour NIHSS score was 8.42±10.63 in the rt-PA group compared to 7.67±8.43 in the urokinase group ($p > 0.05$).<br>(6) The rate of nsICH was 11.54% in the rt-PA group compared to 6.67% in the urokinase group ($p > 0.05$).<br>(7) There were no statistically significant differences in primary and secondary outcomes between the alteplase bridging therapy group and the urokinase bridging therapy group ($p > 0.05$).

AIS, Acute Ischemic Stroke; PIS, Progressive Ischemic Stroke; ESS, European Stroke Scale;NIHSS, National Institutes of Health Stroke Scale; IV, Intravenous; PV, Plasma Viscosity; Fbg, Fibrinogen; CRP, C-Reactive Protein; IL-8, Interleukin-8; Overall response rate (ORR) = (number of cases with complete response + number of cases with partial response + number of cases with stable disease) / total number of cases × 100%; mTIC, Recanalization rate; RCT, Randomized Controlled Trial.

References

e1. Chen, O., He, M., Intravenous thrombolysis with urokinase for acute cerebral infarctions. *Chinese Journal of Neurology* **2002,** *35* (4), 210-213, (in Chinese).

e2. Yuan, Z., Wang, B., Li, F., Wang, J., Zhi, J., Luo, E., Liu, Z., Zhao, G., Intravenous thrombolysis guided by a telemedicine consultation system for acute ischaemic stroke patients in China: the protocol of a multicentre historically controlled study. *BMJ open* **2015,** *5* (5), e006704.

e3. Huang, W., The Efficacy and Safety of Urokinase Intravenous Thrombolysis in Acute Cerebral Infarction. *Electronic Journal of Clinical Medical Literature* **2019,** *6* (39), 23-24, (in Chinese).

e4. Liu, P., LI, X., Gao, Q., Clinical observation of treatment through intrarenous thrombolysis with urokinase for patients with acute cerebral infarction at an early stage. *Chinese Journal of Practical Internal Medicine* **2001**.

e5. Liu, Q., Zhou, H., Fang, Y., Clinical Analysis of Urokinase Intravenous Thrombolysis in the Treatment of 68 Cases of Acute Cerebral Infarction. *Chinese Journal of Practical Nervous Diseases* **2014,** *17* (24), 30-33, (in Chinese).

e6. Li, D., Lei, Y., Shan, S., Influence factors of intravenous urokinase therapy for acute cerebral infarction. *Chinese Journal of Neurology* **2001**.

e7. Yang, L., Guo, B., Effectiveness Evaluation of Intravenous Urokinase Thrombolysis for Acute Cerebral Infarction at Different Time Windows. *Contemporary Medicine* **2017,** *23* (3), 52-53, (in Chinese).

e8. Wang, B., Study on the clinical efficacy of urokinase intravenous thrombolysis in patients with acute cerebral infarction. *China Practical Medicine* **2022,** *17* (3), 1-3, (in Chinese).

e9. Shao, S., Rong, Y., Rong, G.-m., Analysis and Clinical Study of Ultra-early Thrombolytic Therapy for Acute Cerebral Infarction. *Guide of China Medicine* **2020,** *18* (13), 128-130.

e10. Huang, Y., Comparison of the efficacy and safety between urokinase and batroxobin in treatment of acute ischemic stroke. *Chinese Journal of Geriatric Heart Brain & Vessel Diseases* **2006**, (in Chinese).

e11. Su, Q., Su, Q., Chen, S., Liang, Y., Chen, D., Chen, D., Yan, Y., Comparison on efficacy and safety of intravenous thrombolysis with alteplase and urokinase in the treatment of acute cerebral infarction. *China Practical Medicine* **2017,** *12* (30), 1-5, (in Chinese).

e12. Zhou, H., Liu, L., Dong, Z., Effect for intravenous thrombolytic therapy of urokinase combined with recombinant tissue plasminogen activator on DAPK1 and vascular endothelial function in acute cerebral infarction patients. *Sichuan Medical Journal* **2019,** *49* (10), 1050-1055, (in Chinese).

e13. Gao, X., Ren, L., Efficacy and safety of intravenous thrombolysis with rt-PA and urokinase for acute ischemic stroke: Analysis in different time windows. *Journal of Apoplexy and Nervous Diseases* **2019,** *36* (6), 520-523.
e14. Chen, Q., Intravenous thrombolysis with urokinase for acute cerebral infarctions (within 6 h from symptom onset). *Journal of Stroke and Cerebrovascular Diseases* **2001,** *18* (5), 259-61.
e15. Zong, L., Gu, H., Yang, X., Jiang, Y., Li, Z., Wang, C., Trend of Intravenous Thrombolysis and a Comparative Analysis of In-hospital Outcomes of Intravenous Thrombolysis with Alteplase or Urokinase in Acute Ischemic Stroke in China from 2016 to 2022. *Chinese Journal of Stroke* **2023,** *18* (10), 1202-1208, (in Chinese).
e16. Ning, Y., Li, X., Chen, H., The efficacy and safety of intravenous alteplase and urokinase in elderly stroke patients: a secondary analysis of INTRECIS study. *Chinese Journal of Nervous and Mental Diseases* **2023,** *49* (2), 1-5.
e17. Gao, H., Chen, Z., He, G., Chen, F., Lin, H., Chen, R., Lu, Z., Clinical study on valid therapeutic time window of acute cerebral infarction by intravenous thrombolysis with urokinase. *Chinese Journal of Practical Nervous Diseases* **2014,** *17* (23), 75-77, (in Chinese).
e18. Huang, Q., Gu, M., Zhou, J., Jiang, T., Shi, H., Chen, X., Zhang, Y., Endovascular treatment of acute ischemic stroke due to anterior circulation large vessel occlusion beyond 6 hours: a real-world study in China. *BMC Neurol* **2021,** *21* (1), 92.
e19. Yang, L., Efficacy and Safety of Intravenous Thrombolysis with Different doses of Urokinase in the Treatment of Cerebral Infarction. *China & Foreign Medical Treatment* **2016,** *35* (35), 115-117, (in Chinese).
e20. Taylor, B. E. S., Patel, S., Hilden, P., Otite, F. O., Lee, K., Gupta, G., Khandelwal, P., The weekend effect on mechanical thrombectomy: A nationwide analysis before and after the pivotal 2015 trials. *Brain circulation* **2022,** *8* (3), 137-145.
e21. Duan, C., Shen, F., Hou, D., Clinical study on thrombolysis treatment for acute cerebral infarction beyond the standard time window. *China Pharmacist* **2001,** *4* (4), 12-19, (in Chinese).
e22. Wang, R., Gao, L., Exploration of the clinical effect of emergency mechanical thrombectomy for acute large artery occlusion stroke patients with ineffective intravenous thrombolysis using alteplase or urokinase. *Contemporary Medicine* **2022,** *28* (12), 86-88, (in Chinese).
e23. Nong, Y., Bian, Y., Chen, S., Huang, R., Direct Mechanical Thrombectomy versus Bridging Therapy for Acute Large Vessel Occlusion Stroke within 6 Hours of Onset. *Medical Journal of West China* **2022,** *34* (11), 1683-1687, (in Chinese).
